# Chronic Foreign Body Inflammatory Response in Textiloma Formation: Case Report and Analysis of Current Evidence

**DOI:** 10.7759/cureus.102838

**Published:** 2026-02-02

**Authors:** José Serafio-Gómez, Christopher Ernesto Cárdenas Hernández, Diego Jasso Pasillas, José Daniel Pérez Carmona, Mariana Lizbeth Véliz Santana

**Affiliations:** 1 General Surgery, Chihuahua City General Hospital “Dr. Salvador Zubirán Anchondo”, Chihuahua, MEX; 2 Medicine and Surgery, Universidad Cuauhtémoc Campus Aguascalientes, Aguascalientes, MEX; 3 Medicine and Surgery, Universidad Autónoma de Aguascalientes, Aguascalientes, MEX

**Keywords:** abdomen ventral hernia, chronic inflammatory reaction, fibrinous granulomatous reaction, gossypiboma, hypersensibility, infection, polypropylene mesh, surgical complication, textiloma

## Abstract

A gossypiboma (GPB), also referred to as a textiloma (TXT), is a mass formed as a result of the body’s inflammatory and granulomatous response to retained surgical textile material (RSTM), most commonly gauze or mesh fibers. Its reported incidence ranges from 1 in 1,000 to 1 in 10,000 surgical procedures, although the true frequency is believed to be significantly higher due to underreporting related to medicolegal concerns. This entity is clinically important because it may mimic more common postoperative complications such as abscess (ABS), seroma (SER), or mesh infection (MI), frequently leading to diagnostic delay and unnecessary morbidity.

The purpose of this report is to present an uncommon instance of mesh-associated textiloma (MAT) following ventral hernia repair with polypropylene mesh (PPM) and to highlight its radiologic, histopathologic, and clinical features that allow differentiation from other postoperative collections.

A 39-year-old female patient with a history of ventral hernia underwent surgical repair with mesh implantation. Postoperatively, she developed persistent umbilical discharge and, three months later, progressive abdominal fluid leakage. Contrast-enhanced computed tomography (CECT) revealed a well-defined hypodense intraperitoneal collection measuring 80 × 64 × 40 mm with a fistulous tract (FT) extending to the abdominal wall, without evidence of systemic inflammatory dissemination. These features, particularly the encapsulated nature of the collection and the organized fistulization pattern, were highly suggestive of a chronic foreign-body reaction (FBR) rather than an acute ABS, which typically presents with diffuse inflammatory infiltration, or MI, which is associated with diffuse mesh thickening and surrounding cellulitis.

A second surgical exploration was performed, and the resected specimen underwent histopathological examination (HPE), which demonstrated foreign-body granulomatous inflammation (FBGI) with multinucleated giant cells (MGC) surrounding textile fibers embedded in fibrous tissue, consistent with MAT, and without evidence of dysplasia or malignancy. Following complete excision and aponeurotic closure, the patient recovered uneventfully, with no recurrence during follow-up.

This case reinforces that MAT should be included in the differential diagnosis of persistent postoperative collections after mesh-based hernia repair and demonstrates that characteristic CECT findings combined with timely surgical management are essential to reduce morbidity and prevent long-term complications.

## Introduction

Gossypiboma (GPB), also termed "textiloma" (TXT) or "retained surgical textile material" (RSTM), represents a chronic inflammatory and fibrotic reaction triggered by the unintended persistence of non-absorbable textile products such as gauze, surgical sponges, or mesh-associated fibers within the surgical field. Although its estimated frequency ranges from 0.01% to 0.1% of all surgical procedures, this complication remains clinically relevant because it can lead to prolonged morbidity, repeated interventions, and significant medicolegal consequences. From a clinical standpoint, GPB/TXT is important because its presentation is often nonspecific and may closely resemble more common postoperative entities such as ABS, SER, or MI, leading to diagnostic confusion and delayed treatment [1].

Two principal pathophysiological patterns have been described. The exudative inflammatory type results in sterile or infected fluid collections resembling an ABS, whereas the fibrogranulomatous type leads to encapsulation of the foreign material by dense fibrous tissue containing chronic inflammatory cells (CIC) and multinucleated giant cells (MGC), often forming a slowly enlarging mass. The latter is more likely to generate organized FT and well-circumscribed collections, a key radiologic feature that helps distinguish GPB/TXT from SER, which typically lacks thick walls, and from MI, which usually demonstrates diffuse mesh thickening and surrounding soft-tissue inflammation [1].

In the context of abdominal wall reconstruction, polypropylene mesh (PPM) remains widely used due to its high tensile strength, low cost, and availability. However, PPM induces a robust foreign body reaction (FBR) driven by non-biodegradable microfibers, chronic macrophage activation, and, in some cases, low-grade bacterial biofilm formation. These mechanisms promote fibrosis, chronic seroma, mesh extrusion, and enterocutaneous fistula formation. When textile fibers or sponge fragments become trapped within this inflammatory milieu, the development of mesh-associated textiloma (MAT) may occur, as demonstrated in our case [2].

Comparative analysis with previously reported MAT and GPB/TXT cases reveals consistent imaging and pathological patterns. Contrast-enhanced computed tomography (CECT) typically demonstrates a well-defined encapsulated collection, frequently with internal spongiform air bubbles or a whorled internal architecture, and an associated FT extending toward the abdominal wall, notably without evidence of systemic inflammatory dissemination. Histologically, these lesions show foreign-body granulomatous inflammation (FBGI) with MGC surrounding textile fibers, which distinguishes them from ABS (predominantly neutrophilic) and from MI (dominated by bacterial infiltration and mesh degradation) [1].

Given its rarity but significant clinical impact, GPB/TXT remains a highly relevant diagnostic consideration in patients with prior mesh-based hernia repair who present with persistent or atypical postoperative collections. Early recognition through CECT and prompt surgical removal are crucial to avoid chronic infection, fistulization, and repeated failed interventions. Reporting such cases contributes to improved diagnostic awareness, supports better prosthetic selection, and reinforces the importance of strict surgical safety protocols to minimize preventable retained foreign bodies [3].

## Case presentation

A 39-year-old female patient with a history of chronic degenerative disease and recurrent ventral hernia presented with persistent purulent discharge at the umbilical level. She reported having undergone two previous surgical interventions for ventral hernia repair, both with the placement of PPM. The first procedure was complicated by a postoperative SER that was drained without incident, while the second was performed due to persistent discharge from the umbilical scar. Despite these interventions, the patient continued to experience intermittent serous drainage through the umbilicus, associated with a progressively enlarging mass in the same region.

**Figure 1:**
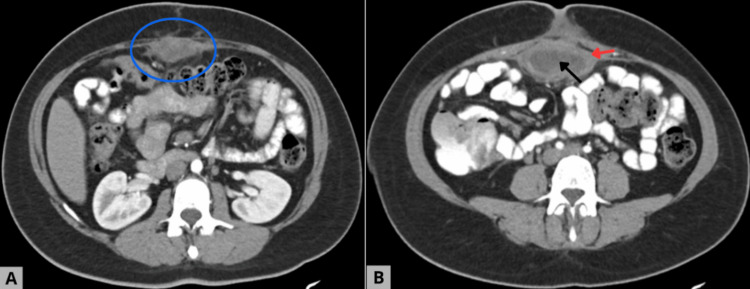
Abdominal computed tomography. The images feature something suggestive of retained surgical foreign material. A. Axial arterial-phase CT image demonstrating a well-defined intraperitoneal mass located along the anterior abdominal wall (blue circle), measuring approximately 80 × 64 × 40 mm. The lesion shows lobulated contours, regular margins, and a heterogeneous internal architecture with a prominent central hypodense area, a characteristic finding highly suggestive of retained foreign material. B. A subsequent CT slice reveals the absence of a discernible separation plane between the mass and the rectus abdominis muscle, indicating firm adherence to the parietal peritoneum. A well-demarcated peripheral inflammatory reaction surrounds the lesion (red arrow), while a distinct central necrotic core is identified (black arrow). The enhancing solid component during the arterial phase (39–50 Hounsfield units) supports an active chronic inflammatory process associated with a foreign-body reaction.

Given the persistence of symptoms, further evaluation was performed. CECT revealed a well-delineated intraperitoneal hypodense collection (Figures 1A, 1B) with a fluid component and an FT extending toward the anterior abdominal wall, without evidence of adjacent abscesses or dissemination. These findings, correlated with the clinical picture, raised suspicion of a complication related to the previously implanted prosthetic material (Figure 1B) [2,4,5].

**Figure 2:**
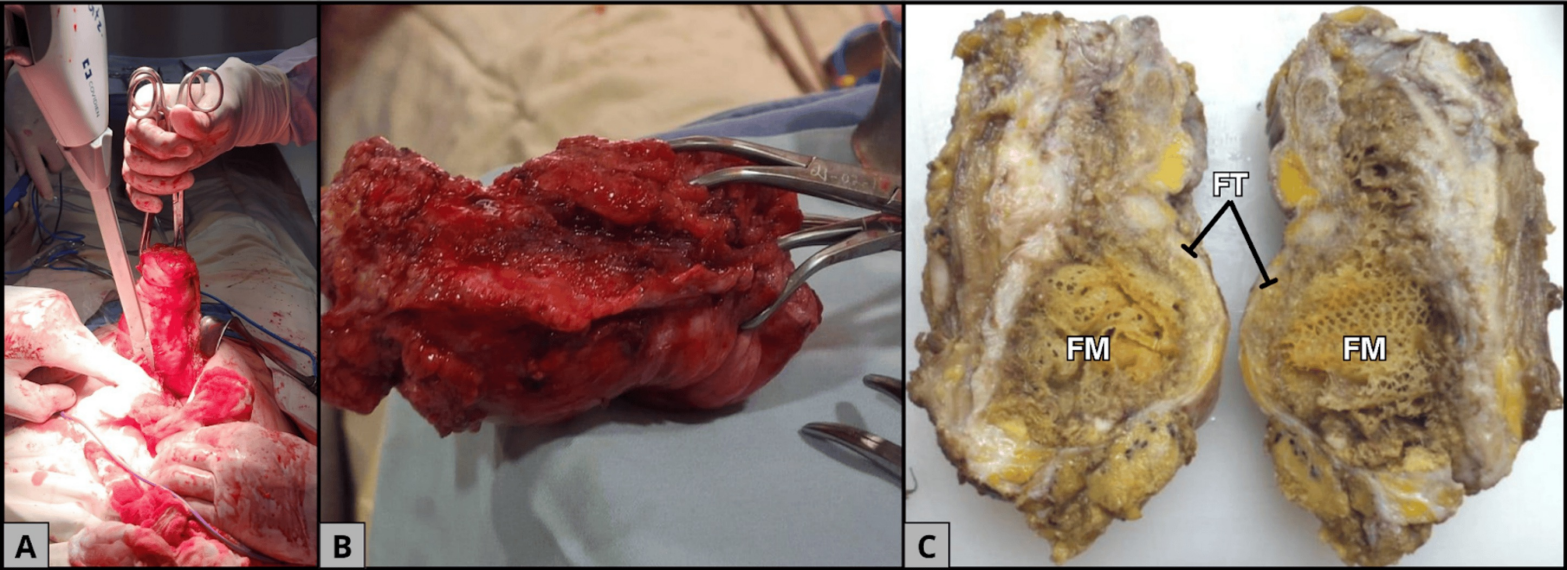
Surgical and gross pathological findings consistent with gossypiboma. A. Intraoperative view showing the extraction of the abdominal mass. B. Intraoperative image of the fully resected specimen. C. Gross examination reveals an ovoid mass measuring 12 × 6 × 5 cm, containing a well-circumscribed whitish-yellow foreign material (FM) consistent with gossypiboma, surrounded by fibroadipose tissue (FT), highlighting the foreign-body reaction.

The patient was scheduled for surgical exploration. Intraoperatively, a well-encapsulated mass adherent to the aponeurosis was identified, containing seropurulent material and degenerated textile remnants, consistent with TXT (Figure 2A). Complete resection of the lesion with surrounding inflammatory tissue was carried out (Figures 2B, 2C), followed by thorough irrigation of the cavity and tension closure of the aponeurosis using absorbable Vicryl sutures [6]. The use of a new polypropylene prosthesis was deliberately avoided due to the demonstrated risk of chronic inflammatory reaction associated with synthetic mesh [1,3].

**Figure 3:**
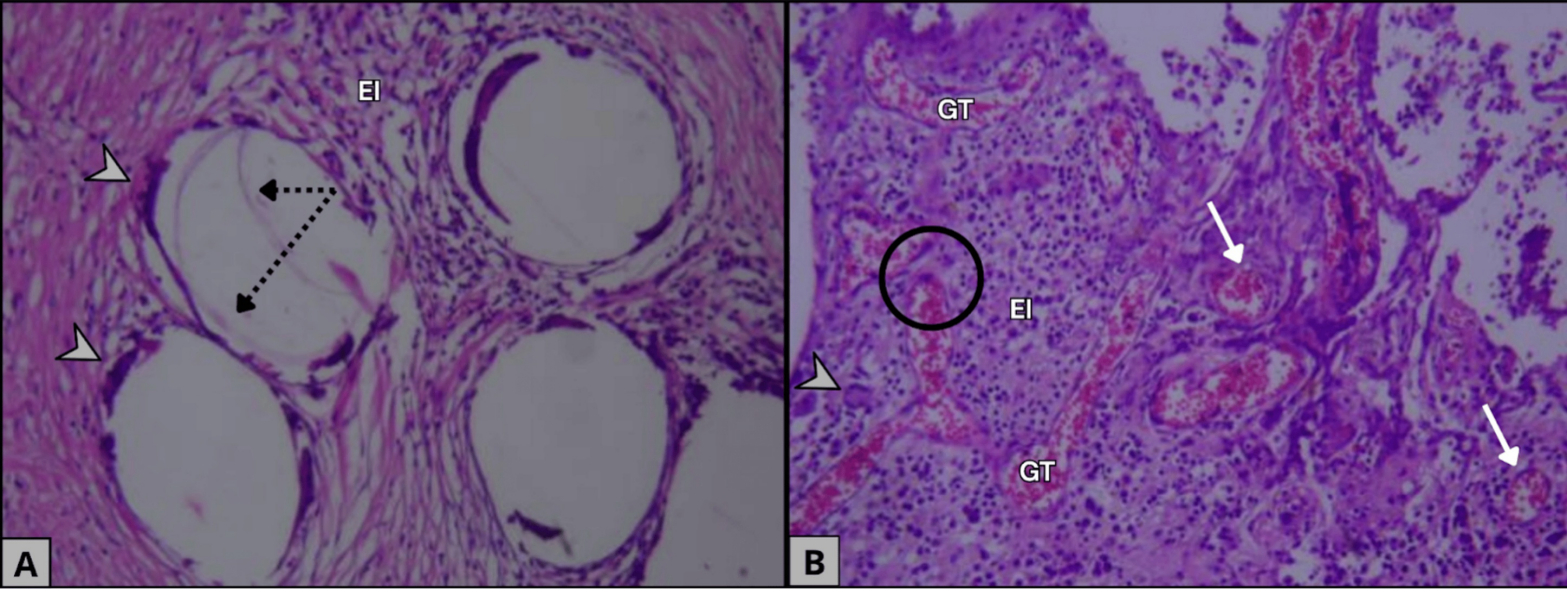
Histopathological features supporting a foreign-body reaction and excluding malignancy. A. Histopathological section showing fibroadipose tissue with fibroblastic and myofibroblastic proliferation consistent with scar formation, along with the presence of foreign textile fibers (dotted arrows) surrounded by a chronic inflammatory infiltrate containing foreign-body–type multinucleated giant cells (arrowheads). B. Histopathological section demonstrating granulation tissue (GT) characterized by thick-walled capillary vessels (white arrows) with preserved endothelial morphology (black circle). A & B. Both sections show areas of edema and inflammatory cell proliferation (EI). The absence of endothelial atypia and the presence of foreign-body–type giant cells support the diagnosis of a foreign-body reaction and confirm the lack of histological evidence of malignancy in the examined specimen.

Histopathological examination (HPE) confirmed the presence of textile fibers surrounded by an intense chronic inflammatory response with fibrosis, lymphoplasmacytic infiltrate (Figure 3A), and multinucleated foreign-body type MGC (Figures 3A, 3B), without evidence of dysplasia or malignancy (Figures 3A, 3B). The postoperative course was favorable, with adequate wound healing, absence of recurrent discharge, and no complications during the early follow-up period.

## Discussion

A textile foreign body is defined as retained surgical material, usually gauze, compresses, or non-absorbable hemostatic agents, that triggers an inflammatory FBR [7]. Its true incidence is difficult to determine because of underreporting and medico-legal implications, but it is estimated to occur in 1 per 1,000-1,500 abdominal operations and up to 1 per 100-5,000 surgical procedures overall [8].

Clinically, the patient presented with intermittent umbilical discharge of a torpid course, accompanied by an abdominal mass that gradually increased in size. These manifestations correspond to the most common presentation of abdominal textileoma (GPB/TXT): a subacute or chronic inflammatory reaction with encapsulation of the foreign body, fistula formation (14.3%), abdominal pain (85.7%), and, in some cases, obstruction (35.7%) or an abdominal mass (28.6%) [9]. In this context, computed tomography (CT) was essential for delineating the intraperitoneal collection and its communication with the abdominal wall, enabling planning of definitive surgical intervention. Notably, the literature describes multiple cases in which imaging findings can be nonspecific and easily confused with tumors or infectious processes, particularly when classic signs such as air bubbles or linear calcifications are absent [9]. This variability reflects the fact that the radiological appearance of GPB/TXT evolves over time and depends on the predominant inflammatory response. In late presentations, the lesion may become more compact and fibrotic, losing characteristic internal architecture and resembling a nonspecific mass or fluid collection. Likewise, linear calcifications are typically associated with chronicity: granulomatous inflammation and the formation of a fibrous capsule favor dystrophic calcification, and calcium deposition may appear as linear or curvilinear streaks along the capsule wall or the folded configuration of the retained material [10].

This complication is not random; therefore, it is essential to consider the factors that increase the risk of GPB/TXT, which can be broadly grouped into those that raise the likelihood of technical error and those that modify the biological response [9].

In the first group, the most relevant factors include emergency surgery, unplanned intraoperative changes, obesity, turnover of operating-room personnel, and multiple procedures performed in the same anatomic region [9]. The latter applied to this patient, who underwent two prior ventral hernia repairs, thereby increasing cumulative operative exposure and the probability that surgical materials may go unnoticed. Reoperations also distort anatomy, promote adhesions, and create more complex operative fields, conditions that collectively heighten vulnerability to retained surgical items [11].

In the second group, the presence of synthetic mesh and previously operated tissue can promote a state of chronic inflammation and/or persistent infection, with manifestations that may arise late, sometimes years after repair. In this context, symptoms such as chronic drainage, pain, or fistulas may be attributed for prolonged periods to “mesh-related complications” or chronic wound infection, which can mask suspicion of a retained foreign body and contribute to delayed diagnosis [12,13]. Moreover, polypropylene mesh placed in contaminated settings or in compromised tissues has been associated with an increased risk of septic complications, underscoring the need to carefully assess the biological environment in the presence of persistent drainage or FT [13].

GPB/TXT can produce two types of reactions: acute and chronic. The acute type manifests a few days after surgery, may involve secondary bacterial contamination, and often follows a septic course ending in abscess formation with possible fistulization to adjacent organs. The chronic type is usually aseptic and may remain asymptomatic for a long time but can become encapsulated, form adhesions, and eventually develop a granuloma [14].

This case underscores the importance of maintaining a high index of suspicion (HIS) for chronic post-surgical symptoms, particularly after complex or repeated interventions in the same anatomical region. Histopathological analysis revealed textile fibers surrounded by intense chronic inflammation with fibrosis and lymphoplasmacytic infiltrate, without dysplasia or malignancy, findings typical of the chronic FBR described in late-presenting GPB/TXT [8].

The case also highlights the need to critically reassess surgical history in patients with persistent or atypical symptoms. In some instances, the time elapsed between the initial operation and symptom onset reduces clinical suspicion, favoring alternative interpretations (e.g., wound infection, granuloma, hernia recurrence) and delaying targeted evaluation [12]. In patients with prior mesh repairs, this effect may be even more pronounced: chronic inflammation or subclinical infection related to the prosthetic material can obscure early manifestations of a retained foreign body, leading to chronic drainage or fistula formation to be initially attributed to “mesh complications” rather than GPB/TXT, thereby contributing to diagnostic delay [12]. 

Institutional and systems-related factors must also be addressed, including standardized surgical count protocols, the routine use of radiopaque-marked sponges, and intraoperative safety systems, since most GPB/TXT cases are preventable events when standardized practices are consistently applied [11]. A notable strength in the management of this case was the surgical decision to avoid implanting new synthetic mesh, given the chronically inflamed environment and the high likelihood of an adverse response if prosthetic material were reintroduced. This approach aligns with reports discouraging mesh use in the setting of active infection or densely inflamed scarring because of the risk of perpetuating inflammation and increasing postoperative complications [13]. Conversely, an important limitation was the prolonged time to definitive diagnosis, which exposed the patient to multiple prior interventions without complete resolution, as well as the psychological and physical burden of a chronic condition that might have been recognized earlier. This outcome reinforces a practical lesson: in patients with persistent fistulas, chronic drainage, or late-appearing masses after abdominal surgery, especially in the setting of reoperations and mesh-early incorporation of retained foreign body into the differential diagnosis and a systematic preventive approach in the operating room are essential to reduce avoidable harm [15].

## Conclusions

This clinical case demonstrates how MAT can manifest as a late and persistent complication of multiple abdominal surgeries, particularly in the context of hernia repair with prosthetic material. Despite its low frequency, this entity should be considered in the differential diagnosis of chronic symptoms such as purulent discharge, mass formation, and FT in patients with a history of surgery.

The clinical evolution of the case highlights the importance of a comprehensive evaluation that includes a detailed surgical history, appropriate use of imaging studies (including CECT), and HPE confirmation, allowing for an effective therapeutic approach. It also reinforces the need for strict preventive measures, such as surgical counting, the use of radiopaque markers, and institutional safety protocols, to reduce the incidence of this preventable complication. This report provides valuable clinical evidence on the diagnostic and therapeutic challenges of GPB/TXT and underscores the relevance of systematic strategies to prevent its occurrence.
